# A Novel Capacitance-Based In-Situ Pressure Sensor for Wearable Compression Garments

**DOI:** 10.3390/mi10110743

**Published:** 2019-10-31

**Authors:** Steven Lao, Hamza Edher, Utkarsh Saini, Jeffrey Sixt, Armaghan Salehian

**Affiliations:** Energy Harvesting and Vibrations Lab, Mechanical and Mechatronics Engineering, University of Waterloo, Waterloo, ON N2L 3G1, Canada; sblao@uwaterloo.ca (S.L.); hedher@uwaterloo.ca (H.E.); utkarsh.saini@edu.uwaterloo.ca (U.S.); jasixt@uwaterloo.ca (J.S.)

**Keywords:** capacitive pressure sensors, in-situ pressure sensing, sensor characterization, physiological applications, cardiac output

## Abstract

This paper pertains to the development & evaluation of a dielectric electroactive polymer-based tactile pressure sensor and its circuitry. The evaluations conceived target the sensor’s use case as an in-situ measurement device assessing load conditions imposed by compression garments in either static form or dynamic pulsations. Several testing protocols are described to evaluate and characterize the sensor’s effectiveness for static and dynamic response such as repeatability, linearity, dynamic effectiveness, hysteresis effects of the sensor under static conditions, sensitivity to measurement surface curvature and temperature and humidity effects. Compared to pneumatic sensors in similar physiological applications, this sensor presents several significant advantages including better spatial resolution, compact packaging, manufacturability for smaller footprints and overall simplicity for use in array configurations. The sampling rates and sensitivity are also less prone to variability compared to pneumatic pressure sensors. The presented sensor has a high sampling rate of 285 Hz that can further assist with the physiological applications targeted for improved cardiac performance. An average error of ± 5.0 mmHg with a frequency of 1–2 Hz over a range of 0 to 120 mmHg was achieved when tested cyclically.

## 1. Introduction

Lower limb compression garments are regularly employed in clinical settings [1] and, more recently, by athletes seeking to improve performance or decrease recovery time [2,3]. In medical settings, compression devices including passive compression socks and active intermittent pneumatic compression devices, are used in the treatment of hypertrophic scarring, venous ulcers, deep vein thrombosis and other diseases [1]. Some examples of physiological testing in [4,5] have shown a significant increase in the mean arterial pressure, cardiac output and cardiac stroke volume, with a significant reduction in the heart rate through applying active compression cyclically at frequencies between 1 to 2 Hz and at pressures of approximately 10 mmHg in the knee region. A major challenge for any compression device, regardless of application, is ensuring the consistency and repeatability of the applied pressure throughout its use [6]. The limited sizing options of most passive compression gear can vary the pressure experienced between subjects due to body shape differences. Further, the consistency of pressure applied by the garment on the same subject can be affected by the donning method [7]. A further challenge is that the shape, size and tissue compliance of the leg can change during compression application due to fluid redistribution, muscle contraction and movement of the garment/device.

Partsch et al. [8] made recommendations on the characteristics that an ideal sensor would need in order to measure the interface pressure between a compression garment and the lower leg skin. Specifically, they identify the need for sensors that are low cost, low hysteresis, have minimal creep, high sampling rate, high accuracy, flexibility, durability and small sensor surface area. To date, there has yet to be a sensor developed that meets all these requirements.

Burke et al. [9] and Ferguson-Pell et al. [10] examined the use of Tekscan FlexiForce and Interlink Electronics FSR piezoresistive-based sensors for measuring interface pressure in compression garments. Piezoresistive sensors function by measuring the change in resistance under a mechanical load. They are thin and can provide reliable results during dynamic measurements. However, these sensors suffer from significant hysteresis and drift, and thus are not dependable for long time-scale measurements [8]. Additionally, piezoresistive sensors are dramatically affected by base curvatures under 32 mm in radius [10] and require relatively large pressures for reliable measurements [11]. The total error exhibited in the sensors, accounting for errors in repeatability, hysteresis and linearity, is approximately +/− 10 mmHg for a pressure range 0 to 96 mmHg, [9], which limits the utility of these devices for pressure mapping of compression garments. Note, for the remainder of the document, unless otherwise noted, errors are described for the pressure range of interest between 0 and 100 mmHg.

Similar to piezoresistive sensors, strain gauges also respond to a change in resistance with mechanical strain [12,13]. Kraemer et al. [14] and Ghosh et al. [15] utilized strain gauges to measure the compression applied by compression garments/bandages on a subject’s thigh and a mannequin leg, respectively. However, since strain gauges exhibit a better sensitivity along their largest dimension, these sensors tend to become thick for measuring interfacial pressures. Additionally, strain gauges tend to exhibit thermal and humidity dependence, and high hysteresis [12].

**Figure 1:**
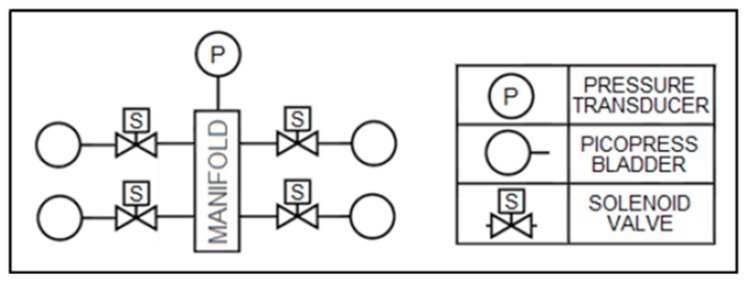
Multiplexing example of four pneumatic sensors.

Pneumatic-based sensors are one of the most prevalent designs for measuring compression garment interfacial pressure [9]. These sensors utilize bladders or compartments that are filled with a small volume of air connected to a pressure transducer through a flexible hose [16]. As pressure is applied, the volume of the bladder decreases, thus increasing the internal pressure. At a constant temperature, it is assumed that the internal pressure measured by the transducer is equivalent to the external applied pressure [16]. Commercially available pneumatic sensors, such as the Salzmann MST MKIV [17], MediGroup Kikuhime [18] and Microlab PicoPress [19] are thin and flexible devices that can be used to measure interfacial pressures between the body and compression device. Furthermore, pneumatic pressure transducers have been found to exhibit repeatable results within +/− 3 mmHg of error [8,19]. A major shortfall of this technology is its temperature dependence [8]. Additionally, their high sensitivity to curvature can result in overestimates of pressure by up to 150% [9]. The sampling rate of the system is affected by the tubing length, which can be important in the evaluation of rapid inflation active compression systems. Additionally, pressure sensing with an array of pneumatic sensors presents multiple inconveniences. The sensors each require a dedicated pressure transducer and have a large surface area (a PicoPress sensor is approximately 50 mm in diameter), resulting in poor spatial resolution. These types of sensors need a great amount of tubing and rely on pressure transducers that are bulky and almost 10 times larger in footprint than the actual sensor, [19]. Sensor multiplexing can reduce the number of transducers in a system, which uses solenoid valves to isolate each bladder for pressure reading (see Figure 1). However, the addition of a manifold increases the system volume, lowering the sampling rate and the transducer’s sensitivity to bladder volume change.

Capacitive sensors have also been employed in other textile applications, [20,21,22], such as muscle activity detection, [20], and pressure mapping for insoles, [21]. These sensors typically consist of a textile used as a dielectric material with conductive yarn woven as electrodes. They have the benefit of being small (good spatial resolution), flexible, and easily used in arrays for pressure distribution measurements [23,24]. Compared to other sensor technologies, they also possess relatively low temperature sensitivities [23,24]. Q. Guo et al. [25] developed a tactile floating electrode capacitive sensor which displayed good linearity up to 350 kPa and improved durability over parallel electrode designs. Capacitive measurements also present their own challenges, often requiring complex circuitry to filter measurement noise [23]. Signal hysteresis can also affect performance, as the dielectric material is often viscoelastic and exhibits nonlinear behaviour when stressed [26]. Additionally, the current-pressure relation is nonlinear [27] which makes it more challenging for proper calibration. Nano-fibers have been employed in the development of pressure sensing technologies for other applications [27,28]. However, due to the high temperature sensitivity of materials used, such as gold, they are not suitable for the application of interest. 

In this paper, a novel, capacitive-based, dielectric electroactive polymer (DEAP) pressure sensor is developed in collaboration with StretchSense Ltd. for in-situ pressure measurements under textile garments. Specific testing conditions are required when evaluating pressure sensors for compression garments, since the interface is neither flat nor solid. Thus, multiple testing methods are presented in Section 3, with results in Section 4. The sensor’s pressure sensitivity, error and durability are characterized. The performance effects due to temperature, humidity, boundary conditions and hysteresis are also examined. This is the first sensor to use DEAP materials measuring low pressure ranges (0 to 100mmHg) for portable and wearable applications, including the fast sampling rate required for many physiological applications. In addition to the sensor fabrication and characterization, the small, portable wireless circuitry for the proposed sensor is one of the novel aspects of this work. The wireless sensing unit communicates data over Bluetooth with the actuation system.

## 2. Capacitive Sensors

**Figure 2:**
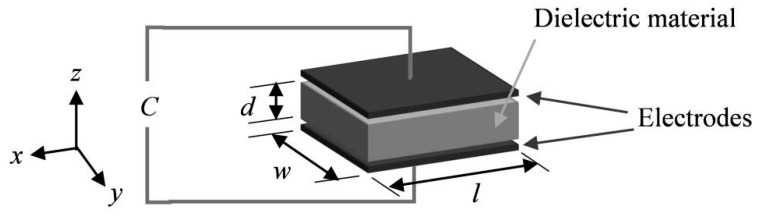
Parallel plate capacitor model of the pressure sensor.

The custom designed sensors developed in collaboration with StretchSense Ltd. for this research are capacitive tactile sensors operating on the principle of a deformable parallel plate capacitor model (shown schematically in Figure 2). The dielectric layer is made of a silicone-based DEAP. The model consists of two compliant electrode layers of length l and width w separated by a dielectric material of thickness d forming a compliant capacitor [29].

The capacitance *C*, for a parallel plate capacitor is described by:

(1) $$C=\varepsilon_{r}\varepsilon_{0}\frac{lw}{d}$$

where $\varepsilon_{r}$ and $\varepsilon_{0}$ are the relative permittivity of the dielectric layer and the vacuum permittivity respectively. Compression of the capacitive sensor along the *z*-axis, (see Figure 2), causes a decrease in the dielectric layer thickness and the distance between the two electrodes, *d*. Assuming unconstrained edges and an incompressible material for the dielectric layer, this results in an increase in the area, (lw), [26]. Both changes result in an increase of the capacitance, which can then be correlated to an applied pressure.

**Figure 3:**
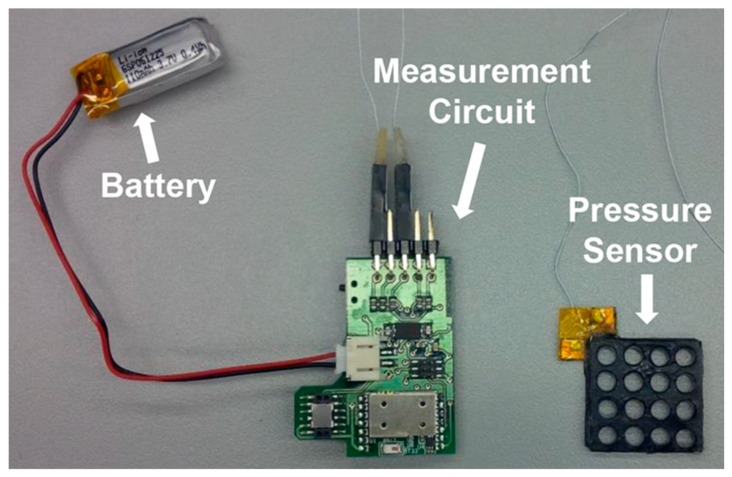
StretchSense pressure sensor, wireless measurement circuit, and battery.

**Figure 4:**
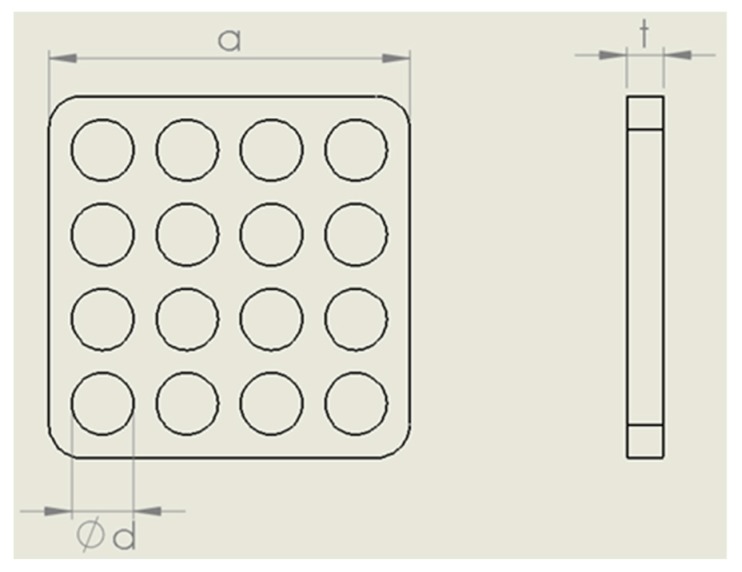
StretchSense pressure sensor schematic.

A picture of the capacitive sensor used in this study is shown in Figure 3. This is the third generation of the sensor developed by the authors, which has better sensitivity, increased sampling frequency, wider moving average filter, improved manufacturing procedure and improved noise rejection. The system consists of a battery-powered circuit capable of measuring the capacitance of five sensing elements. There is a trade-off with respect to sensor head size, as the change in capacitance with pressure is directly proportional to the sensor area, while the spatial resolution reduces with increasing size. In order to maintain a small footprint, the outer surface of the sensor is electrically insulated and folded onto itself like an accordion to increase the capacitor area. This folding of the sensor effectively creates a multi-layer sensor, where each layer experiences the same pressure, resulting in a higher initial capacitance and improved pressure sensitivity. As evident in Figure 3, the sensor head is perforated with a series of holes. These holes decrease the overall stiffness of the sensor, allowing greater deformation during compression. Figure 4 shows a simplified schematic of the pressure sensor with major dimensions labeled. Table 1 provides the dimensions and elastic modulus of the StretchSense pressure sensor, as well as specifications of the battery used. The elastic modulus was averaged between two sample tensile tests at a strain rate of 12 mm/minute and is applicable for strains below 10%. In comparison to pneumatic sensors, the sensor design used is only ~25% of the PicoPress bladder surface area. This provides a significant advantage in terms of the design’s spatial resolution.

The capacitance of the sensor is sampled at 660 Hz by a measurement circuit made by StretchSense. The circuit then applies a moving average of 20 data points to generate a final output at 285 Hz. A merit of using capacitive sensing is the stability of sampling rates that are largely independent from experimental setup conditions. Conversely, pneumatic sensor sampling rates are more susceptible to setup conditions as discussed in Section 1. This high sampling rate is particularly useful for the physiological applications where more dynamic pressure applications, such as pulsations tuned at a person’s heart rate, are used to increase the cardiac performance [4,5]. The control circuitry used is designed to minimize noise while maximizing the output data rate.

### Comparison to Alternative Capacitive Sensors

The unique DEAP construction presents multiple advantages compared to many parallel plate and floating electrode constructions. Since the electrodes are made of a compliant material with similar stiffness to the silicone dielectric layer, interlayer stresses during bending and stretching are minimized. This construction avoids using thin traces that are common for many parallel plate structures, which may break under repeated bending loads, as also noted by M-Y Cheng et al. [30]. Additionally, a major advantage of the DEAP parallel plate construction used is its simplicity and resistance to fail under many cycles of loading. The fabrication of the floating electrode and parallel plate sensors is often more complex, requiring multi-step micromachining processes to create air gaps and metal layers [25,31]. Conversely, the sensor presented avoids such process steps during fabrication. This greatly improves manufacturing scalability and reduces design complexity.

When compared to the DEAP parallel electrode construction used, Q. Guo et al. [25] presented a floating electrode design using a robust construction and good linearity over a wider pressure sensing range (up to 350 kPa, or 2625 mmHg). However, the target application of medical compression garments where pulsations are used requires much lower pressure ranges, (0–100 mmHg). Within the scope of these smaller signals, sensor noise and error reduction becomes increasingly critical for the target design. M-Y Cheng et al. [30] showed that while theoretical sensitivities of parallel plate and floating electrode constructions are the same, parallel plate constructions are predicted to have a better signal-to-noise ratio (SNR). Thus, for the application’s low-pressure operating range, the parallel plate construction used is more advantageous to maximize SNR.

## 3. Experimental Methods

**Figure 5:**
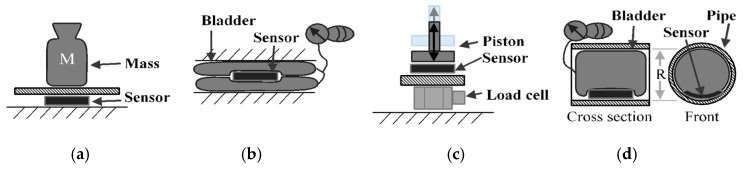
Test configurations for pressure sensor validation. (**a**) Mass Test, (**b**) Bladder Test, (**c**) Piston Test, (**d**) Curvature Test.

In this section, the test procedures assessing sensor performance in representative configurations are outlined. One of the challenges in assessing in-situ pressure sensor performance is the difficulty in applying a realistic and measurable (known) boundary (loading) condition. Research that includes the measurement of pressures applied to the leg often neglects the measurement errors introduced when moving from laboratory testing facilities to in-situ measurements. For example, sensor calibration is typically performed on a flat, solid surface; the effect of curvature and compliance of the leg are not examined [16]. Therefore, one of the objectives of this section is to study the errors that are introduced when taking in-situ pressure measurements on a leg. For this purpose, a series of representative tests were formulated to examine the behaviour of these capacitance-based sensors in different modes. Figure 5 depicts four test setups used to validate the sensor. The tests in all configurations were repeated several times to gather relevant statistics.

**Figure 6:**
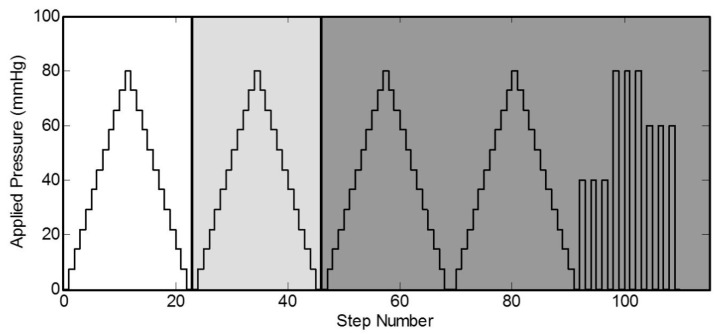
Testing protocol for applied load on sensor in the static tests.

The mass test (Figure 5a) is performed on a flat surface where the sensor sits with masses stacked on top of it. The mass applied increases in 50 g increments from 0 g up to 550 g. The applied pressure to the sensor is determined by measuring the total weight placed on the sensor over the area of the sensor. The sensor is subjected to static loads at increments of approximately 7 mmHg that are held for 30 s at a time, up to a maximum pressure of 80 mmHg. The average and standard deviation of the capacitance for a 10 s period is collected for each increment. The 10 s period is taken after the load has been applied and the measurement reading has settled. The loading profile (shown in Figure 6) has four incremental loading and unloading cycles and nine direct loading and unloading cycles. The incremental cycles are used to measure the hysteresis inherent in the sensor. Meanwhile, the direct loading cycles closely match the use-case for these sensors when applied under, for example, intermittent pneumatic compression devices. The results from the first incremental loading and unloading cycle (white section in Figure 5) are discarded as a “bedding-in” cycle, as recommended by StretchSense, to allow the sensor to acclimate to a stable position. The second incremental loading and unloading cycle (light grey section) is then used to calibrate the sensor. A fit is applied to the calibration data and this curve fit is then used to convert the capacitance measurements of the remaining validation data (dark grey section) to pressure estimates. This test is meant to examine repeatability, linearity and hysteresis of the sensor in static conditions.

Figure 5b depicts the bladder test, whereby the sensor is placed between two inflatable (18 cm × 38 cm) bladders. The bladders are inflated by a hand pump up to a pressure of 110 mmHg following the same timing procedure as used in the mass test, while the air pressure is measured using a manometer. The assumption is that the internal pressure of the bladder is transmitted to the pressure on the sensor. The main purpose of this test is to mimic a condition where the sensor rests on a soft surface and is used as additional verification for the previous test. It should be noted that the sensor is extremely lightweight, weighing only 1.28 g, as stated in Section 2. As such, the weight of the sensor per area is negligible compared to the pressures of interest. 

In the piston test (Figure 5c), the sensor is placed on top of a dynamic load cell (PCB Piezotronics 208C01, Depew, NY, USA) and an impulse is applied to the unit by a pneumatic cylinder. The pneumatic cylinder is controlled with a pressure regulator and flow restrictor valve to enable dynamic pressure application at 1–2 Hz. In this test, the peak values from the load cell and the sensor measurements in each cycle of the pneumatic cylinder were compared. Similar to the mass test, the pressure being applied to the sensor is simply the force measured by the load cell over the area of the sensor. With this test, the dynamic response and repeatability over 400 loading cycles was tested at peak pressures ranging from 0 to 120 mmHg.

The fourth test (Figure 5d) is used to determine the change in behaviour due to the curvature of the sensor. The curvature is expected to change the response of the sensor since it deforms when it conforms to a curved surface and, therefore, the capacitance changes. In the curvature test, the sensor is placed inside pipes of 19.9 mm and 39.9 mm radii, and a balloon is inserted and inflated to apply pressure in the range of 0 to 110 mmHg. The static pressure of the balloon is compared to the sensor reading, similar to the bladder test. It is noted that the curvature in this test remains constant and one of the surfaces is rigid, which is not the actual use case. However, this configuration was selected to isolate the effect of sensor curvature.

**Figure 7:**
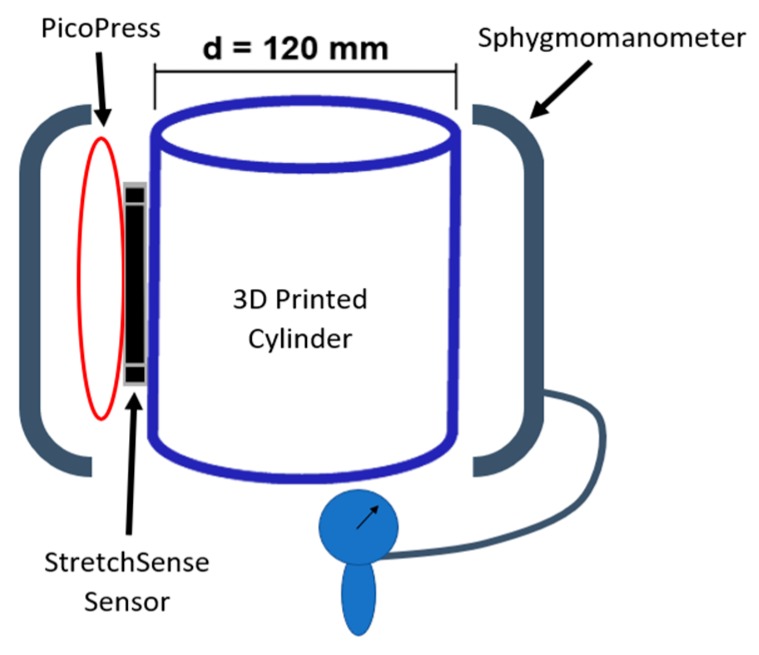
PicoPress and StretchSense pneumatic comparison test.

Finally, two additional testing configurations not shown in Figure 5 are employed. First, the effect of temperature and humidity on the no-load capacitance of the sensor was examined. This is done by repeating the mass test at 26 °C and then comparing it to the data from the 29 °C mass test. In addition, the variance in capacitance of the sensor is observed for a range of 17 to 42 °C and 45 to 69 %RH. Second, a curvature test compares the response of the capacitive sensor to that of the PicoPress pneumatic sensor. This test involved a 3D printed cylinder with a radius of 60 mm to serve as a rigid dummy leg (see Figure 7). The two sensors are placed on the cylinder with the StretchSense sensor against the cylinder and the PicoPress directly on top of it. Since the PicoPress sensor is considerably larger in size than the StretchSense sensor, the entirety of the StretchSense sensor contacts the PicoPress bladder. A sphygmomanometer is then wrapped around the cylinder and sensors to apply a known pressure. The pressure in the sphygmomanometer is manually controlled with the hand pump. The test involves a series of 10 inflations and deflations of the sphygmomanometer at three different pressures. The applied pressures are 40 mmHg, 60 mmHg, and 80 mmHg, respectively. During deflation, a minimum pressure of 5 mmHg is kept in the sphygmomanometer to prevent the cuff from moving and/or slipping. In this test, the sensor responses were both compared to the sphygmomanometer pressure and each sensor error were calculated.

## 4. Results and Discussion

The tests described in the previous section were performed on the capacitive pressure sensor presented in Section 2. The results for each test are presented in this section.

### 4.1. Mass Test

**Figure 8:**
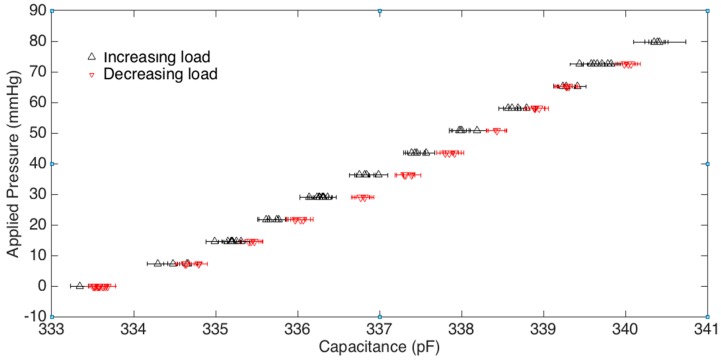
Sensor capacitance at various applied pressures–mass test results.

The results of the mass test are shown in Figure 8, where the mean capacitance measurement is plotted along with error bars for each increment. The error bars indicate +/− twice the standard deviation of the measurement at each point, which gives a 95 % confidence that values lie between the bars. The graph is plotted with the capacitance on the x-axis since the interpretation of the sensor would require the reading of the capacitance to determine the pressure applied. The slope of a linear fit through all the data points (loading and unloading) indicates that the sensor sensitivity is 0.0847 pF/mmHg. The average error bar width is +/− 0.12 pF, which corresponds to an average pressure error of +/− 1.4 mmHg when converted using the sensitivity defined above.

**Figure 9:**
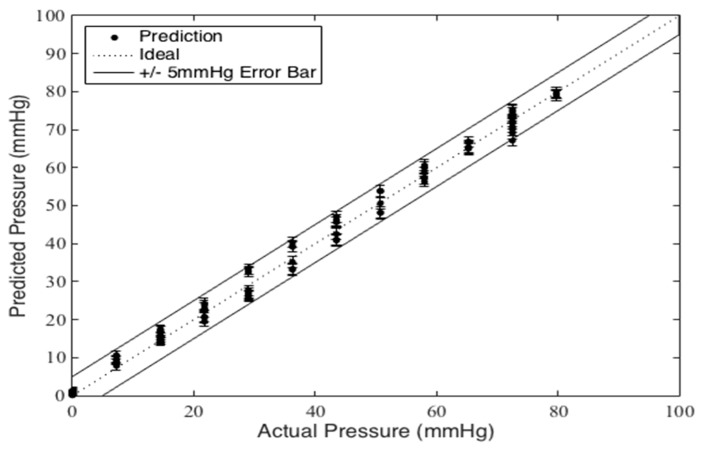
Predicted pressure compared to actual applied pressure in mass test.

There is a discrepancy between measurements when the masses are being loaded versus unloaded, indicating that there is hysteresis in the system. The hysteresis error is defined as half of the span of the capacitance at a given pressure from loading versus unloading. The data points with increasing load are shown with triangles pointed upwards, while the decreasing loads are shown with triangles pointed downwards. The average hysteresis error across the applied pressures was computed to be +/− 3.0 mmHg. The overall error of the sensor in this test setup is just outside +/− 5.0 mmHg as also shown in Figure 9 which displays the predicted pressures versus the actual pressures using the aforementioned sensitivity calibration. As shown in this figure, the percentage error is significantly smaller for the upper range of pressure.

### 4.2. Bladder Test

**Figure 10:**
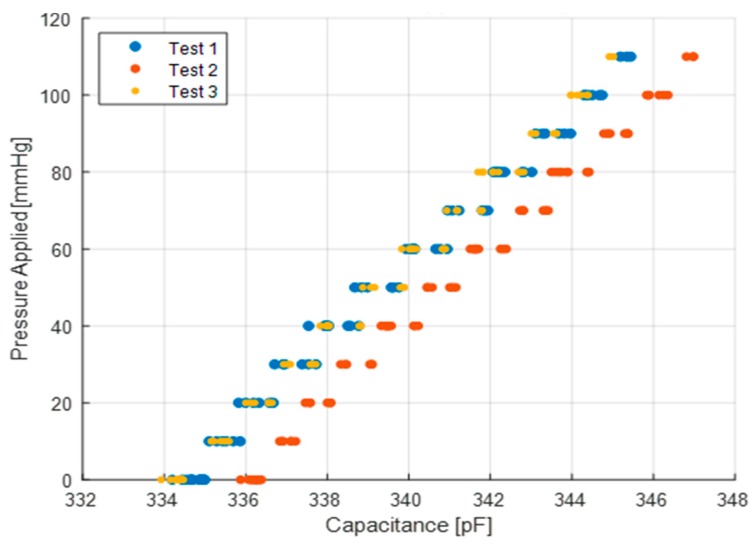
Sensor capacitance at various applied pressures–bladder test results.

The bladder test described in Section 3 was repeated three times and the results are shown in Figure 10. To ensure repeatability, the setup was disassembled and reassembled after every test. The pressure was applied using a sphygmomanometer of +/− 3 mmHg accuracy. The test results show an offset of 2 pF in one of the tests for all pressure ranges shown, however, it is noteworthy that the sensor sensitivity for the three tests remains the same. The causes for the offset are explored in the coming section that examines the effect of temperature and humidity on the sensor performance. The sensor sensitivity from the bladder test calibration was found to be 0.100 pF/mmHg, which is approximately 18% higher than the results from the mass test. This is likely due to the softer material in contact with the sensor during these tests that allows for larger expansion along the plane of the sensor, thus increasing its deformation. On a hard, rigid surface, the sensor sticks to the surface and the friction restricts deformation. In the case of the mass test, the rigid surface in contact with the sensor ultimately results in a smaller sensitivity for similar pressure values. The average measurement noise is approximately +/− 0.18 pF (+/− 1.8 mmHg).

### 4.3. Piston Test

**Figure 11:**
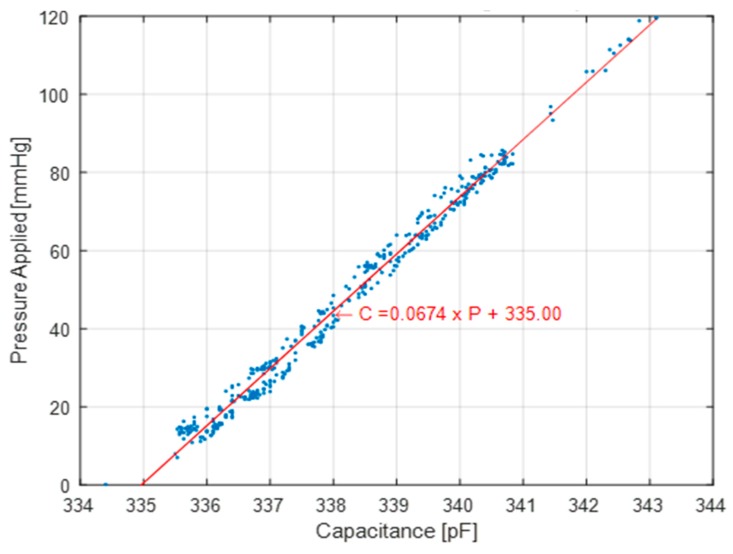
Capacitance change with dynamic pressure application–piston test results.

The piston test setup is designed to examine the repeatability of the sensor readings over the course of over 400 loading and unloading cycles. Figure 11 presents the peak values of the load cell pressure readings versus the capacitance of the pressure sensor. The degree of vertical scatter in these data points for a given capacitance represents the repeatability of the sensor measurements, which is considerably smaller at larger pressure values. The hysteresis is not captured during this test since only the maximum load at each cycle is recorded. This results in a smaller overall error of +/− 5.0 mmHg when measuring the vertical scatter in the plot. The best curve fit for Figure 11 corresponds to a sensitivity value of 0.067 pF/mmHg which is on the same order of magnitude as the mass test sensitivity.

**Figure 12:**
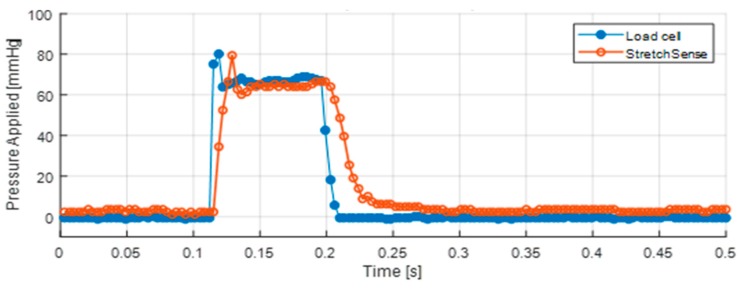
Dynamic response of load cell versus StretchSense sensor during one piston load.

The temporal signals during one piston stroke are presented for the load cell and capacitive sensor in Figure 12. A sharp peak is noted at the start of the signal, which is attributed to the initial impact of the piston against the sensors. This impact is disregarded in the analysis. In order to allow for a comparison between the load cell and the StretchSense measurements, the capacitance was converted to pressure using a linear curve fit as the calibration. As shown in this figure, the magnitudes of the calibrated StretchSense signal and the load cell are in good agreement. The negligible 10–20 ms latency in the capacitive sensor measurements is expected, given the data smoothing done by the measurement circuit board. 

The samples did not show signs of structural damage, including on the electrical connections, after applying more than 400 loading and unloading cycles. This is believed to be due to the perforated geometry and soft sensor material, which allows for compliance in the structure. This allows the sensor to perform better under fatigue loads, in comparison to a solid geometry. Electrical connections are also insulated and reinforced with Kapton tape to improve durability.

### 4.4. Curvature Test

**Figure 13:**
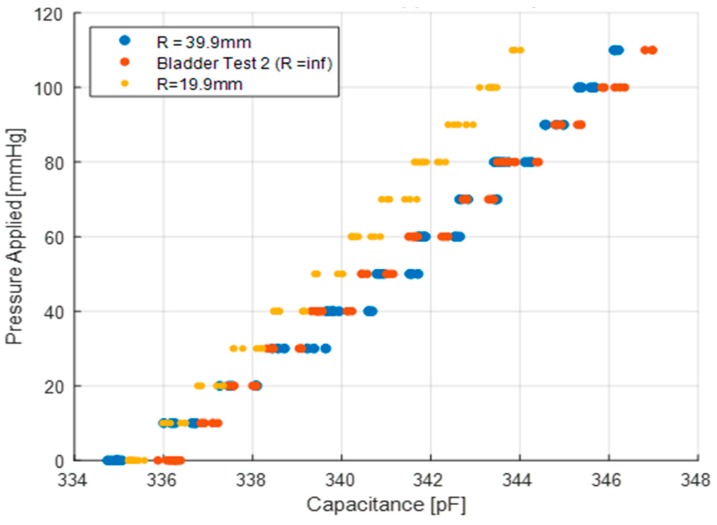
Pressure vs sensor capacitance for various radii of curvature.

The sensitivity to curvature of the capacitive sensors is shown in Figure 13 for a variety of pressures. The curvature clearly influences the sensor capacitance when subject to pressure. The sensitivity of the sensor was found to be 0.0812 pF/mmHg and 0.106 pF/mmHg for radii of curvature of 19.9 mm and 39.9 mm, respectively. From the bladder test, the sensitivity on a flat surface (infinite radius) is 0.100 pF/mmHg. As shown in this figure, the sensor sensitivity decreases as the radius of the curvature decreases, i.e., the sensor is more curved. This sensitivity to curvature may potentially impose limitations in the sensor calibration when mounted on a flexible object of variable curvature, such as a calf muscle. This can be mitigated if the sensor is securely placed against a rigid plate of fixed curvature attached to the user. The sensors may then be calibrated where the plate closely fits a person’s local body shape during in-situ measurements. Of course, a rigid plate changes the pressure loading experienced by the compression user.

### 4.5. Thermal and Humidity Effects

As shown in Section 4.2, an offset of up to 2 pF was observed between several test runs, which relates to a 20 mmHg variation across the pressure range for these tests. This is a significant source of error, particularly for the smaller pressure values. It is reasoned that the mechanical setup of the fixture should be tolerant to changes in orientation due to the conformity of the air bladders employed in the bladder test. To further examine the source of this offset value shown in Figure 10, the effect of temperature and humidity is studied.

**Figure 14:**
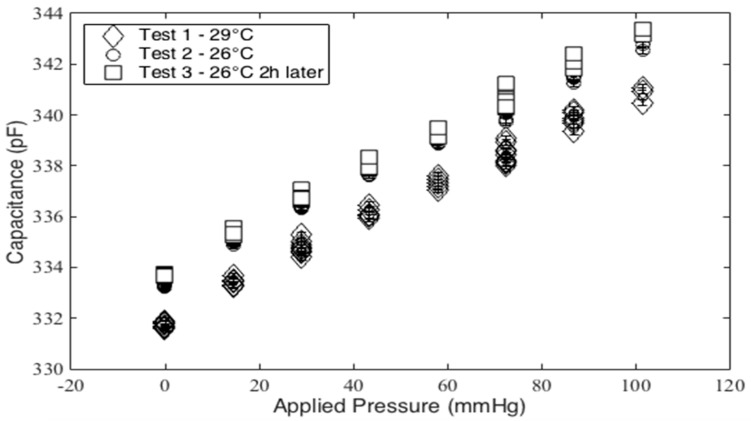
Capacitance vs applied pressure for various temperatures.

In initial testing in the mass test setup, the temperature of the sensor was allowed to settle at a temperature of 29 °C and the test was performed. The temperature was then dropped to 26 °C and the mass test was repeated twice over an approximately two-hour period. As shown in Figure 14, there is a notable offset with the change in temperature. Like the bladder test results shown in Figure 10, the sensitivity of the sensor is the same despite the offset. This offset is termed the no-load offset. 

**Figure 15:**
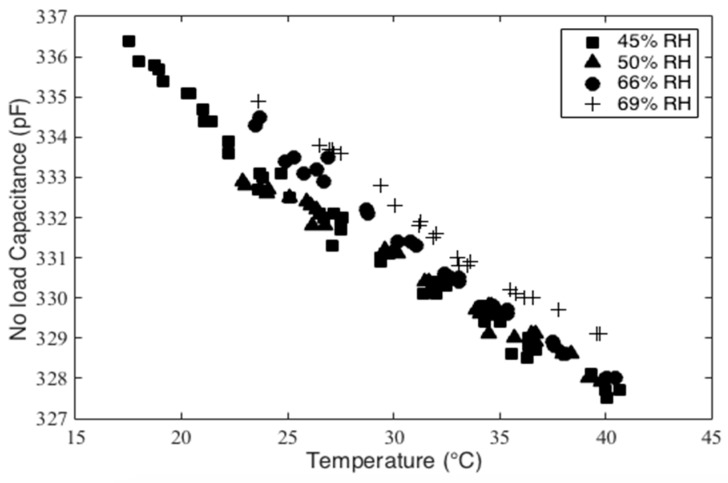
No load capacitance versus temperature and humidity.

Further tests were performed to characterize the offset by measuring the capacitance under the no-load condition while varying temperature and humidity. The sensor was placed on a Peltier module, which acts as a hot/cold plate in order to control the sensor temperature. The capacitance was then measured at several ambient humidity and temperature values, as shown in Figure 15. Using the experimental results shown in this figure, the sensor’s temperature sensitivity, or thermal coefficient, was found to be approximately −0.38 pF/°C, and the humidity sensitivity was measured to be −0.345 pF/%RH. This relates to approximate pressure errors of 4 mmHg/°C and 4 mmHg/%RH. These tests indicate that both the temperature and humidity have noticeable effects on the sensor’s output. Thus, the variances in the bladder test results are likely attributed to the differences in the temperature and humidity conditions during these tests. It was noted, however, that the ambient conditions were not recorded prior to bladder testing.

The mathematical proof shown below explains the reason behind the offset created by temperature. It should be noted that it may be assumed that the permittivity remains fairly constant for the given temperature range. This is ultimately the reason for the sensitivity (slope of the pressure-capacitance plot) remaining unchanged for various temperatures. Therefore, assuming $\varepsilon_{r}$ remains constant, any change in temperature, *T*, results in changes of thickness, length and width of the dielectric layer as shown below:

(2) $$C_{T_{0}}=\varepsilon_{r}\varepsilon_{0}\frac{lw}{d}\to C_{T}=\varepsilon_{r}\varepsilon_{0}\frac{l\left(1+\alpha \Delta T\right)w\left(1+\alpha \Delta T\right)}{d\left(1+\alpha \Delta T\right)}=\varepsilon_{r}\varepsilon_{0}\frac{lw\left(1+\alpha \Delta T\right)}{d}=C_{T_{0}}\left(1+\alpha \Delta T\right)$$

(3) $$C_{T}=C_{T_{0}}+C_{T_{0}}\alpha \Delta T$$

Here, $C_{T}$ is the capacitance for temperature T, and $C_{T_{0}}$ denotes the capacitance for temperature $T_{0}$. The above equation shows that a given temperature change, ΔT, results in a constant offset $C_{T_{0}}\alpha \Delta T$ compared to its reference value at $T_{0}$. Therefore, with permittivity constant, the pressure-capacitance relation has the same slope for all temperature values, and the temperature change only causes a capacitance offset. It should also be noted that the DEAP material has a negative coefficient of thermal expansion which results in a negative offset upon an increase of temperature as shown in Figure 15.

### 4.6. Pneumatic-Based Sensor Comparison

**Figure 16:**
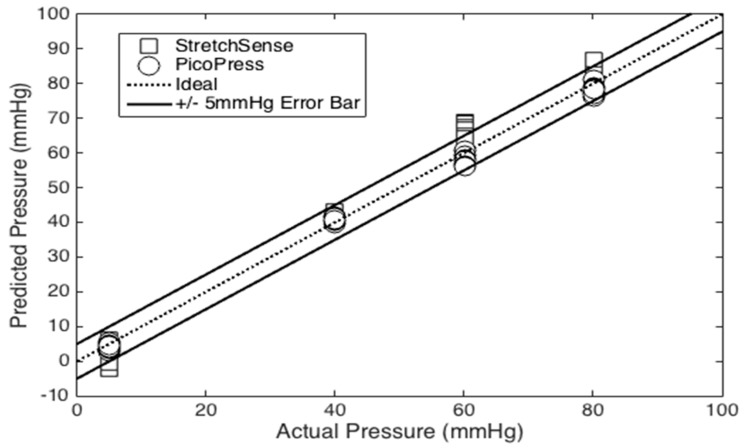
Experimental comparisons of StretchSense and PicoPress sensors.

The results of the pneumatic sensor comparison tests are plotted in Figure 16. The linear sensitivity value obtained during the bladder test was used to calibrate the StretchSense sensor, since those test conditions were most-similar to the cylindrical test bed setup. The results show that the PicoPress exhibits an average error of +/− 6.38 mmHg. Meanwhile, the StretchSense sensor exhibits an average error of +/− 8.03mmHg. The error is calculated by averaging the sum of the sensor calibration error and twice the standard deviation for each pressure value. As described in earlier sections, these errors include the influence of hysteresis, measurement noise, curvature and biases in calibration. Decreasing the capacitive sensor footprint can help to reduce curvature sensitivity.

From the tests performed, recorded sensor sensitivities and errors have been grouped in Table 2 for reference. The sensitivity and error are both seen to vary depending on the testing method implemented. The piston test reported the lowest sensitivity, and the pneumatic sensor comparison test returned the largest error observation. As a result, to perform a conservative characterization of similar sensors in the future, the piston and pneumatic sensor comparison tests should be used to determine sensor sensitivity and error, respectively.

## 5. Conclusions

This paper assesses the feasibility of a capacitance-based DEAP sensor technology for tactile pressure measurement in compression garment applications. The sensor is the outcome of a few generations of design iterations between the academic and industry partners. A series of tests were introduced to evaluate the effects of various factors on reliable in-situ pressure sensing. For the wide range of tests presented, the sensor has shown to have errors varying from ±1.8 mmHg for the bladder test to ±8.03 mmHg for the pneumatic sensor comparison test. The variation of the sensor sensitivity in response to mounting curvature was assessed. For sensors of smaller footprints, this becomes less significant. Temperature and humidity were studied and quantified for their impact on a bias error for the no-load capacitance value. The sensor calibration can easily help mitigate this as the sensitivity was shown not to be affected by these variables.

Compared to a pneumatic sensor, some of the advantages of the DEAP capacitive sensor design are its superior spatial resolution, manufacturability for smaller footprints, as well as its overall compact and simple design for use in array configurations measuring pressure distributions. This sensor performs at a high sampling rate of 285 Hz to allow measurements of abrupt pressure changes for physiological applications where a pressure pulsation may need to be tuned to a person’s heartrate in order to increase cardiac output. The sensor is thin enough to be worn under a compression garment (thickness of 2.25mm). The durability shown during cyclic testing is attributed to the sensor being made of one solid component and no gaps within the sensor, as well as the compliance of the polymer solution used. Additionally, the perforated design will make the sensor more compliant to help prevent crack initiations during cyclic loading. Future sensor development can potentially improve performance, such as enhanced noise filtering using the electronics. As well, sensor geometry optimization should be further investigated using Finite Element Analysis to optimize the sensor’s performance for the desired range of pressures. This includes investigating the trade-off between smaller sensor footprints, which achieves better spatial resolution and smaller curvature errors, but reduces the sensor’s overall sensitivity.

## Figures and Tables

**Table 1:** StretchSense pressure sensor and measurement equipment details.

Property	Value
Sensor width, *a*	22 mm
Sensor hole diameter, *d*	3.75 mm
Sensor thickness, *t*	2.25 mm
Sensor weight	1.28 g
Sensor elastic modulus	0.78 MPa
Battery weight	3.04 g
Battery capacity	110 mAh
Battery voltage	3.7 V

**Table 2:** Sensor sensitivity and error values for each test.

Test Type	Sensor Sensitivity (pF/mmHg)	Sensor Error (mmHg)
Mass Test	0.085	± 5.0
Bladder Test	0.100	± 1.8
Piston Test	0.067	± 5.0
Curvature Test (19.9 mm radius)	0.081	N/A
Curvature Test (39.9 mm radius)	0.106	N/A
Pneumatic Sensor Comparison–PicoPress Sensor	N/A	± 6.4
Pneumatic Sensor Comparison–StretchSense Sensor	N/A	± 8.0
